# The Glial Scar-Monocyte Interplay: A Pivotal Resolution Phase in Spinal Cord Repair

**DOI:** 10.1371/journal.pone.0027969

**Published:** 2011-12-21

**Authors:** Ravid Shechter, Catarina Raposo, Anat London, Irit Sagi, Michal Schwartz

**Affiliations:** 1 Department of Neurobiology, The Weizmann Institute of Science, Rehovot, Israel; 2 Department of Biological Regulation, The Weizmann Institute of Science, Rehovot, Israel; University of Nebraska Medical Center, United States of America

## Abstract

The inflammatory response in the injured spinal cord, an immune privileged site, has been mainly associated with the poor prognosis. However, recent data demonstrated that, in fact, some leukocytes, namely monocytes, are pivotal for repair due to their alternative anti-inflammatory phenotype. Given the pro-inflammatory milieu within the traumatized spinal cord, known to skew monocytes towards a classical phenotype, a pertinent question is how parenchymal-invading monocytes acquire resolving properties essential for healing, under such unfavorable conditions. In light of the spatial association between resolving (interleukin (IL)-10 producing) monocytes and the glial scar matrix chondroitin sulfate proteoglycan (CSPG), in this study we examined the mutual relationship between these two components. By inhibiting the *de novo* production of CSPG following spinal cord injury, we demonstrated that this extracellular matrix, mainly known for its ability to inhibit axonal growth, serves as a critical template skewing the entering monocytes towards the resolving phenotype. *In vitro* cell culture studies demonstrated that this matrix alone is sufficient to induce such monocyte polarization. Reciprocal conditional ablation of the monocyte-derived macrophages concentrated at the lesion margins, using diphtheria toxin, revealed that these cells have scar matrix-resolving properties. Replenishment of monocytic cell populations to the ablated mice demonstrated that this extracellular remodeling ability of the infiltrating monocytes requires their expression of the matrix-degrading enzyme, matrix metalloproteinase 13 (MMP-13), a property that was found here to be crucial for functional recovery. Altogether, this study demonstrates that the glial scar-matrix, a known obstacle to regeneration, is a critical component skewing the encountering monocytes towards a resolving phenotype. In an apparent feedback loop, monocytes were found to regulate scar resolution. This cross-regulation between the glial scar and monocytes primes the resolution of this interim phase of spinal cord repair, thereby providing a fundamental platform for the dynamic healing response.

## Introduction

Every year, spinal cord injury (SCI), a debilitating condition with a limited prognosis for recovery, paralyzes around 130,000 people. The poor recovery of the central nervous system (CNS), a delicate tissue that cannot tolerate toxic conditions, is generally attributed to the hostile local milieu created at the trauma site. Two major barriers to repair that have been identified include the local inflammatory response, acknowledged for its neurotoxic potential, and the creation of the glial scar, known to impair regeneration [1], [2], [3]. The axonal growth inhibitory effects of the scar matrix were supported by numerous *in vitro* studies demonstrating that such molecules cause neurite retraction and growth cone collapse [4], along with their well-defined developmental role in formation of boundaries. Accordingly, research efforts and clinical manipulations were directed at attempts to eliminate and reorganize the chemical components of the glial scar [5], [6] and to suppress the ensuing immune response [7]. Recent studies, however, indicated that the scar and some immune cell populations each have independent, though transient, positive roles. The glial scar was shown to provide an ‘SOS’ response, a distress signal initiated by the tissue in response to the trauma that demarcates the lesion site and restores the isolation of the CNS from the circulation [8], [9]. Likewise, leukocytes were demonstrated to promote removal of tissue debris, secrete neurotrophic factors, and support axonal regeneration [10], [11], [12], [13], [14], [15].

Recently, a pivotal role for recovery was attributed to monocytes that infiltrate the damaged CNS due to their non-classical anti-inflammatory/resolving properties [11], [14]. These cells were shown to produce the anti-inflammatory cytokine, interleukin 10 (IL-10) and to terminate the local microglial response. Based on their inflammation-resolving properties, these monocyte-derived cells correspond to the previously identified macrophage subset with immunoregulatory properties, the resolving/regulatory macrophages (rMΦ), observed in wound healing [16], [17], or myeloid derived suppressor cells (MDSC), which occur in cancer. Comparable suppressive monocytes were identified also in other pathologies, including myocardial infarction [18], [19]. Advances in the field of myeloid cells, revealing macrophage heterogeneity and monocyte plasticity, brought this often neglected population back into the spotlight. Until now, the main factor determining the phenotype of the monocytes was thought to be the surrounding cytokine milieu [16], [17], [20]. While a pro-inflammatory milieu, enriched in either IFN-γ or TNF-α, skews monocytes towards a classical pro-inflammatory (M1) phenotype, a Th2/anti-inflammatory environment, composed of IL-4 and IL-13, or IL-10 and TGFβ, generates alternatively-activated (M2) or rMΦ, endowed with healing properties [11], [14]. Given the pro-inflammatory environment at the site of trauma [21], in the current study, we aimed to identify the factors that maintain the healing properties of the infiltrating monocytes under such pro-inflammatory conditions. In light of the immunomodulatory effects recently attributed to the glial scar matrix component, chondroitin sulfate proteoglycan (CSPG), in microglial education [9], together with the spatial association between the infiltrating monocytes and this glial scar component [9], we investigated here the mutual regulation between these two key processes in the course of the response to injury. We show that CSPG is an essential platform, skewing the infiltrating monocytes towards their resolving anti-inflammatory phenotype. In an apparent reciprocal loop, the monocyte-derived cells acquire matrix-degrading properties enabling their resolution of the glial scar; this function was found to be critically dependent on their expression of the matrix metalloproteinase 13 (MMP-13), thereby creating a more permissive environment for axonal regeneration. This scar remodeling function of the infiltrating monocyte-derived cells reveals a novel and crucial property of these essential cells, which was found to be fundamental to recovery, by resolving not only inflammation but scar deposition, as well.

## Results

### Resolving macrophages are embedded in a pro-inflammatory milieu and are confined to the region of glial scar deposition

**Figure 1:**
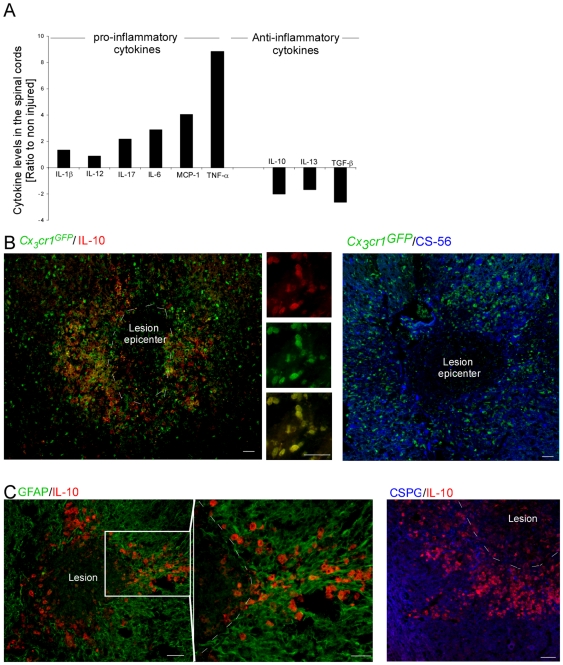
Resolving macrophages are restricted to a region enriched with glial scar matrix. (**A**) *Luminex* analysis of the cytokine profile at the injured spinal cord. The results are presented as the ratio of expression levels relative to non-injured animals. Pooled samples (n = 3) were analyzed. The results are presented as change relative to the non-injured tissue. One representative experiment is shown out of two repetitions, each conducted at three different time points during the first week post injury (d1,3,7). The same tendency was observed for each time point tested, and in each repetition. The injury skews the local environment towards a pro-inflammatory milieu. (**B**) Spinal cord sections of injured [*Cx_3_cr1* ^GFP/+^>wt] BM chimeras, isolated at day 7 post injury, were co-stained for the infiltrating monocytes by GFP (green), and for the anti-inflammatory cytokine, IL-10 (red), or the glial scar matrix component, CSPG (CS-56; blue). (**C**) Injured spinal cord sections, isolated at day 7 post injury, co-stained to reveal the glial scar (astrocytes appear in green, and CSPG matrix protein in blue) and IL-10 (red), showing that resolving macrophages (rMΦ) are restricted to the CSPG-enriched area. Scale bar; 50 µm.

Since the cytokine milieu is a major determinant of the differentiation fate of monocytes [17], [20], [22], we tested the cytokine profile that these cells encounter when reaching the injured spinal cord parenchyma. To this end, we examined the cytokine profile at the lesion site during the first week post injury. Pooled spinal cord tissues, 4 mm in length that included the lesion site, margins and surrounding undamaged parenchyma, were homogenized and freeze-thawed to extract the proteins. The extracts were tested for production of cytokines using a Multiplex system that simultaneously analyzes an array of cytokines in the same sample. Multiplex analysis of M1/M2-skewing cytokines revealed that following trauma to the CNS, the local environment at the lesion site becomes biased towards a pro-inflammatory milieu, dominated by the most characteristic cytokine that determines the M1 skewing, TNF-α (**Fig. 1A**). The same tendency was observed at all tested time points, and for all repetitions, but the fold change varied. Immunostaining of spinal cord sections for IL-10, a predominant M2- skewing cytokine, revealed its basal expression by neurons of the healthy tissue, its downregulation following injury, and its specific induction at later time points by macrophages surrounding the lesion site (**Figure S1**). The constitutive expression of IL-10 by neurons, and the loss of this expression following injury, explains the small post injury reduction seen by the Luminex analysis, which was reproduced using an ELISA specific to IL-10.

Since these results suggest that the cytokine milieu was unlikely to account for the differentiation of the infiltrating monocytes to rMΦ, we hypothesized that other predominant factor(s) are likely to play a fundamental role in this process. Matrix molecules influence immune cell behavior during autoimmune disease [23], and robust alterations in the extracellular matrix are observed in the traumatized CNS [24]; thus, in light of the recently identified immunomodulatory role of CSPG, the predominant extracellular component of the glial scar matrix that endows microglia/macrophages with neuroprotective properties characterized by their production of insulin like growth factor 1 (IGF-1) [9], we assessed the contribution of this matrix to monocyte skewing towards their essential resolving properties.

Although the monocyte-derived macrophages and activated resident microglia are functionally distinct, there is currently no differential morphological marker that can distinguish between them. Thus, in the present study, we used a well-established bone marrow (BM) chimera model [25], in which the BM of irradiated (using head shielding) host mice, is replaced in adulthood by genetically labeled BM expressing green fluorescent protein (GFP) under the control of the myeloid promoter, *Cx_3_cr1* [26], enabling the clear distinction of infiltrating monocyte-derived macrophages (GFP^+^) from resident microglia (non-fluorescent), as reported previously [14], [27]. The mice were analyzed for their chimerism 8 weeks following transplantation, and were subjected to spinal cord injury. Immunohistochemical analysis of the injured spinal cord parenchyma 7 days post injury revealed that the skewed monocyte-derived cells (Cx_3_cr1^GFP^) that acquired a rMΦ phenotype (demonstrated by the high expression of the anti-inflammatory cytokine, interleukin (IL)-10, the hallmark of this subset [17], as previously shown [14], and as verified here **Fig. 1B**), were found to concentrate at areas enriched with the glial scar-matrix molecule, CSPG (**Fig. 1C**). As was previously demonstrated [14], no resolving IL-10 producing monocyte-derived macrophages accumulated in the epicenter of the lesion, despite the abundant accumulation of other macrophages there; notably, this area is devoid of scar tissue.

### The glial scar matrix molecule, chondroitin sulfate proteoglycan, determines the resolving phenotype of the monocytes that encounter it

The spatial association between the glial scar matrix CSPG and the infiltrating monocyte-derived cells, in light of the immunomodulating properties attributed to this scaffold [9], prompted us to test whether this matrix is involved in the immune-skewing of the infiltrating monocyte-derived cells towards their resolving, anti-inflammatory phenotype. Using *de-novo* inhibition of CSPG biosynthesis via the administration of the pharmacological inhibitor xyloside, previously used in both *in vitro* and *in vivo* studies [28], we have previously shown that CSPG is fundamental for the repair following spinal cord injury, when restricted to the acute phase post-injury [9]. In the same study, we suggested that this matrix modulates the macrophages/microglia that encounter it to attain non-cytotoxic neuroprotective properties, characterized by reduced TNF-α and increased IGF-1. In our previous study we noted that such treatment disrupted the spatial organization of the monocyte-derived cells relative to the injury site; however, the impact on the phenotype of these cells was not addressed. As we found that the resolving monocyte-derived macrophages were concentrated at the lesion margins in association with CSPG deposition, we next tested whether the same *in vivo* strategy for inhibition of CSPG biosynthesis would affect not only the location of these cells but also their phenotype.

**Figure 2:**
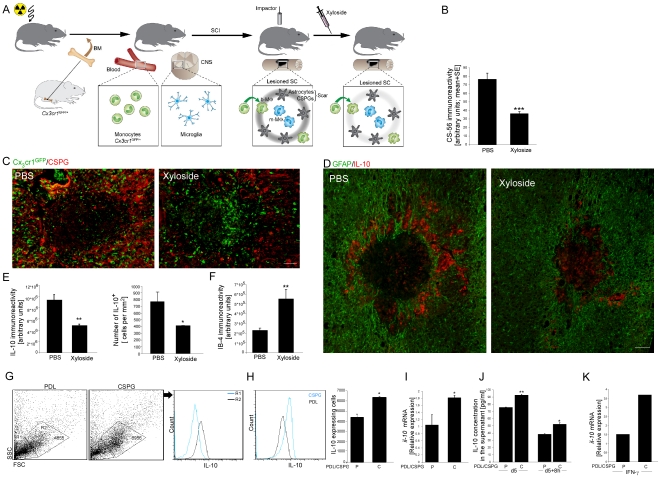
The glial scar component chondroitin sulfate proteoglycan determines the resolving/anti-inflammatory phenotype of the encountering monocytes. (**A**) Schematic illustration showing the experimental design. Spinal cord-injured [Cx3cr1^GFP/+^>wt] BM chimeras were subjected to spinal cord injury 8 weeks following BM transplantation. Immediately post injury, mice were treated with PBS or xyloside, an inhibitor of CSPG production, twice a day for 5 consecutive days. (**B**) Quantification of CSPG immunoreactivity at the lesioned spinal cord following treatment with xyloside, as assessed immediately after the last injection (*Student's t-test*; ***p<0.001). (**C**) Labeling of spinal cord sections for detection of CSPG (red) and GFP (green). Inhibition of CSPG synthesis disrupted the spatial compartmentalization of the infiltrating monocytes (GFP^+^), which are now located at the epicenter of the lesion. (**D**) Representative pictures of IL-10-expressing cells (red) and their location relative to the lesion epicenter, demarcated by GFAP expression (green), at day 7 post injury. Xyloside treatment abolished expression of this anti-inflammatory cytokine at the margins. (**E**) Quantitative analysis of IL-10 immunoreactivity (left panel, *Student's t-test*; **p = 0.004) and number of IL-10 expressing cells (right panel, *Student's t-test*; *p = 0.04), at day 7 post injury, in 2 mm^2^ isolated tissue sections, including lesion site, margin and surrounding parenchyma. (**F**) Quantification of activated microglia/MΦ according to IB-4 immunoreactivity (*Student's t-test*; **p = 0.007), as measured at day 14 post injury in 2 mm^2^ tissue sections, including lesion site, margin and surrounding parenchyma. (**G–K**) *In vitro* cultures of naïve CD115^+^ monocytes seeded on poly-D-lysine (PDL) or CSPG-coated flasks (3–4 cultures per group were analyzed in each experiment). The results presented are representative of several independent experiments performed. (G, H) Flow cytometric analysis for intracellular expression of IL-10. Two populations that differed in their size and granularity were identified (R1, R2), expressing differential levels of IL-10. CSPG seeded monocytes became enriched with the R2 population, which expressed higher levels of IL-10 (*Student's t-test*; *p = 0.025). (**I, J**) Cultures were harvested for analysis of *Il10* gene expression by Real-Time PCR (I; *Student's t-test*; *p = 0.05), and the supernatants analyzed by ELISA for IL-10 protein expression (J; *Student's t-test*; **p = 0.0021; following replacement of media *p = 0.015). (**K**) Higher *Il10* mRNA expression was observed in the CSPG coated dish even in the presence of IFN-γ, indicating that CSPG is a strong inducer of the resolving phenotype, even under pro-inflammatory/M1-skewing conditions. Scale bar; 50 µm. *y*-axis error bar represents SEM.

To that end, [*Cx_3_cr1* ^GFP/+^>wt] BM chimeric mice, created following irradiation and reconstitution with labeled BM, were subjected to spinal cord injury 8 weeks post transplantation, and were treated twice a day for 5 consecutive days with either PBS or xyloside, starting immediately following the contusion (**Fig. 2A**). In line with our previous report [9], such treatment with xyloside, which led to a 50% reduction in CSPG deposition at the lesion margins when analyzed immediately after the last xyloside injection (**Fig. 2B**), resulted in monocyte infiltration into the epicenter of the lesion at day 7 post injury, an area from which they were excluded in the presence of CSPG (**Fig. 2C**). Notably, the changes in the spatial compartmentalization of the infiltrating GFP monocytes were not accompanied by any alteration in their cell numbers (*Student's t-test*; p = 0.365). Yet, this early inhibition of CSPG production had a negative impact on their acquisition of an anti-inflammatory phenotype, manifested by reduced IL-10 expression at day 7 post injury, as quantified using the *ImagePro* software on 2 mm^2^ antibody-labeled spinal cord sections that included the lesion site, margins and surrounding parenchyma (**Fig. 2D, E**). We next tested whether this reduction in IL-10 expression was associated with a suppression of the activated microglial response, as previously described by us [14]. We found that the reduction of the suppressive potency of the monocyte-derived cells was accompanied by enhanced activation of the resident microglia, as indicated by IB-4 immunoreactivity at day 14 post injury, and its evaluation by *ImagePro*, as above (**Fig. 2F**). The fact that partial inhibition of CSPG had a dramatic effect on the resolving phenotype of the infiltrating monocyte-derived cells suggests that this matrix has a fundamental biological significance in determining the fate of these cells. As the extracellular matrix around the site is a complex branched structure, it is likely that such partial inhibition has dramatic effect on the local organization of the perineuronal network created around the lesion site following injury. It is probable that the complex structure limits the spread of the toxic material concentrated at the epicenter, and prevents the infiltrating cells from coming into contact with this milieu enriched with M1- skewing cytokines. In this manner, even partial inhibition would breach this matrix capacity to serve as a structure insulating the two compartments from each other.

Notably, the observed alterations following xyloside treatment were not due to non-specific effects of the drug, as slightly delayed administration of xyloside, starting at day 2 post injury (for 5 consecutive days), as was previously shown [9], did not lead to increased microglial activation, nor did it affect the spatial organization of the infiltrating monocytes. In addition, such delayed administration did not affect IL-10 production by these monocytes (**Figure S2**). As the levels of IL-10 immunoreactivity at the site measured on day 7 post injury were not affected by the delayed application of xyloside, the reduced activation of the resident microglia, as indicated by IB-4 immunoreactivity at day 14 post injury, could not be attributed to the anti-inflammatory nature of the infiltrating monocytes. We thus suspect that the delayed inhibition has other effect(s) that result in such IB-4 regulation.

As the injured spinal cord is known to contain a large amount of myelin debris, factors that were previously shown to have modulatory M1/M2 effects following their engulfment by macrophages [29], [30], we next tested whether such myelin uptake might be responsible for monocyte skewing towards their IL-10-expressing phenotype. Phagocytosis by macrophages of degradation products of myelin was tested using Oil Red O (ORO) staining, as previously described [10], [31]. ORO staining of spinal cord sections taken at day 7 post injury revealed equal distribution of macrophages that engulfed myelin in the lesion epicenter and at its margins (**Figure S3**). This uniform distribution of macrophage uptake of myelin was not in spatial correlation with the resolving, IL-10 producing phenotype of the macrophages, and thus was not likely to participate in their skewing. To verify that the observed effect seen following immediate xyloside treatment could not be attributed to changes in myelin engulfment, we ORO stained spinal cord sections isolated from either PBS or xyloside- treated mice (day 7 post injury). Although significant reduction was observed in IL-10 immunoreactivity following xyloside treatment, no noteworthy differences were observed between the two groups in the myelin engulfment by macrophages. This suggests that while an M1/M2 modulating effect has been attributed to myelin engulfment [29], [30], the acquisition of the resolving phenotype by the monocyte-derived cells at the injured spinal cord, characterized by the expression of the anti-inflammatory cytokine IL-10, does not seem to be related to uptake of myelin.

To reveal the direct effect of CSPG on monocyte skewing, we employed an *in vitro* assay using primary cultures of naïve CD115^+^ monocytes seeded on CSPG or on an inert substrate, Poly-D-lysine (PDL), as a basal reference. Flow cytometric analysis of the cultured cells showed that these substrates induced the development of two populations that differed in their morphology (based on size and granularity) as well as in their IL-10 expression levels (**Fig. 2G**). However, the CSPG cultures became enriched with the population comprised of cells expressing higher levels of IL-10 (R2; **Fig. 2G, H**). Similarly, increased overall IL-10 expression was observed in CSPG-cultured monocytes (**Fig. 2I, J**). Enhanced expression of this anti-inflammatory cytokine was also observed in the presence of IFN-γ, a potent M1-skewing factor (**Fig. 2K**), suggesting that the glial scar matrix plays a dominant role determining the phenotype of the monocytes that encounter it, even in a pro-inflammatory setting. Thus, our data demonstrate that the glial scar matrix molecule, CSPG, is a critical immunoregulatory scaffold, inducing the monocytes towards the rMΦ subset, characterized by their production of the anti-inflammatory cytokine, IL-10.

### Infiltrating monocytes promote glial scar matrix resolution via the production of matrix metalloproteinase 13

The well recognized matrix degrading properties and tissue remodeling of MΦ as part of peripheral wound healing [32], and especially of the resolving monocyte-derived cells [18], [19], prompted us to examine whether the infiltrating monocytes are not only affected by CSPG, but might in turn regulate the resolution of this scar matrix molecule, which is known to be a major obstacle for CNS regeneration in the chronic phase [3], [5], [33].

**Figure 3:**
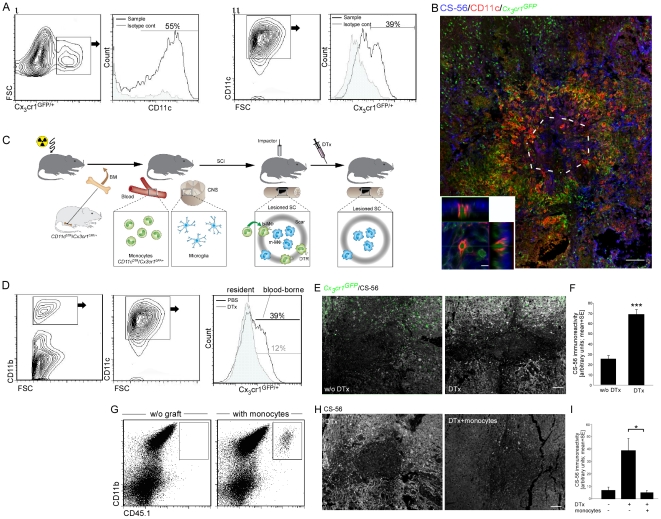
Infiltrating monocytes resolve glial scar matrix accumulation. (**A**) Flow cytometry analysis of spinal cord tissues isolated from spinal cord injured [*Cx_3_cr1* ^GFP/+^>wt] BM chimeras at day 7 post trauma. The histograms were pre-gated according to the presented topography plot, following gating on CD11b^+^ cells. GFP and CD11c expression are approximately 50% correlated. (**B**) Representative confocal micrograph of longitudinal sections isolated at day 7 post injury from injured spinal cord of [Cx3cr1^GFP/+^>wt] BM chimeras, labeled for CS-56 (blue), GFP (green), and CD11c (red). Lower panel: *z*-axis projection of a single cell. (**C**) Schematic illustration of the experimental design: [*CD11c*-DTR:*Cx_3_cr1* ^GFP/+^>wt] BM chimeras were subjected to SCI, 8 weeks post BM transplantation; half of them received DTx. (**D**) Flow cytometric analysis of cells from the lesion site of DTx-treated and non-treated [*CD11c*-DTR: *Cx_3_cr1* ^GFP/+^>wt] BM chimeras, demonstrating depletion of CD11c-expressing monocytes but not of their resident counterparts, the microglia. The histograms were pre-gated according to the presented topographic plots. (**E, F**) Labeling of the spinal cord tissues sections, isolated at day 14 post injury, with CS-56 (white). Quantitative analysis of CSPG immunoreactivity is presented. Depletion of monocytes by DTx dramatically increased CSPG accumulation (*Student's t-test*; ***p = 0.0001). (**G–I**) Spinal cord injured [CD11c-DTR:Cx_3_cr1^GFP/+^>wt] BM chimeric mice were treated with DTx and were adoptively transferred with wt CD115^+^ monocytes (resistant to DTx treatment). Control groups included chimeric animals without monocyte transfer, with and without DTx treatment. (G) Flow cytometric analysis of the spinal cord lesion site with or without adoptive transfer of CD115^+^ monocytes (CD45.1^+^) following DTx treatment. (H,I) Spinal cord tissues sections, isolated at day 14 post injury, labeled with CS-56 (CSPG; white), with and without reconstitution of *wt* monocytes is presented in H. Quantitative analysis of CSPG immunoreactivity is presented in I. Depletion of monocytes dramatically increased CSPG accumulation, which was prevented by adoptive transfer of naïve monocytes (ANOVA; F_2,10_ = 5.46; *p* = 0.05). Scale bar: (B) 100 µm (bottom panel; 10 µm); (E,H) 50 µm. *y*-axis error bar represents SEM.

To this end, we adopted an *in vivo* cell ablation strategy that targets the monocyte-derived cells in close proximity to the glial scar matrix. Approximately 50% of the monocytes infiltrating the lesioned spinal cord were found to be CD11c^+^ at day 7 post injury (**Fig. 3A**), and those CD11c^+^ *Cx3cr1* ^GFP^ monocyte-derived cells resided at the margins of the site in close association with CSPG enriched areas (**Fig. 3B**). We therefore used a previously employed [14] conditional *in vivo* cell ablation strategy targeting the monocyte-derived cells in virtue of their CD11c promoter activity [34]. Specifically, we generated [*CD11c*-DTR: *Cx_3_cr1* ^GFP/+^> wt] BM chimeras, using head protection during irradiation, as previously described [14]; GFP expression in the transferred cells allowed us to trace the infiltrating monocytes, and the Diphtheria Toxin Receptor (DTR) transgene enabled us to specifically deplete this cell population upon their upregulation of CD11c (**Fig. 3C**). The chimeras were tested for their chimerism 8 weeks following the BM transplantation, and then were immediately subjected to spinal cord contusion, and treated with Diphtheria Toxin (DTx). As previously reported, such treatment resulted in the specific depletion of GFP^+^ cells, corresponding to the infiltrating monocytes, without affecting their CNS counterparts, the resident microglia (GFP^−^) (**Fig. 3D**). DTx-dependent depletion of monocyte-derived cells in close proximity to scar deposition resulted in a higher level of CSPG immunoreactivity, as evaluated on day 14 post injury, the peak of CSPG accumulation, using computerized *ImagePro* analysis of images of 2 mm^2^ spinal cord sections (**Fig. 3E,F**). We restored the monocyte pool of DTx-treated chimeras by intravenous injection of wt CD115^+^ monocytes (carrying the allotypic marker, CD45.1; injected on d0 and d3 post injury) that did not harbor the *CD11c*-DTR transgene, and whose descendants were therefore resistant to the toxin treatment. The monocytes infiltrated the injured spinal cord (**Fig. 3G**; as previously reported [14]), and were found to be sufficient to restore the lost regulation of the glial scar matrix deposition observed in the depleted mice (**Fig. 3H,I**). Accordingly, while DTx-treated chimeras showed massive accumulation of CSPG around the lesion epicenter at day 14 post injury, reconstitution of DTx-treated mice with monocytes resistant to the depletion resulted in decreased accumulation of this scar matrix component. These results demonstrate a novel aspect of the resolving properties of the recruited monocytes associated with the resolution/termination of the glial scar deposition.

**Figure 4:**
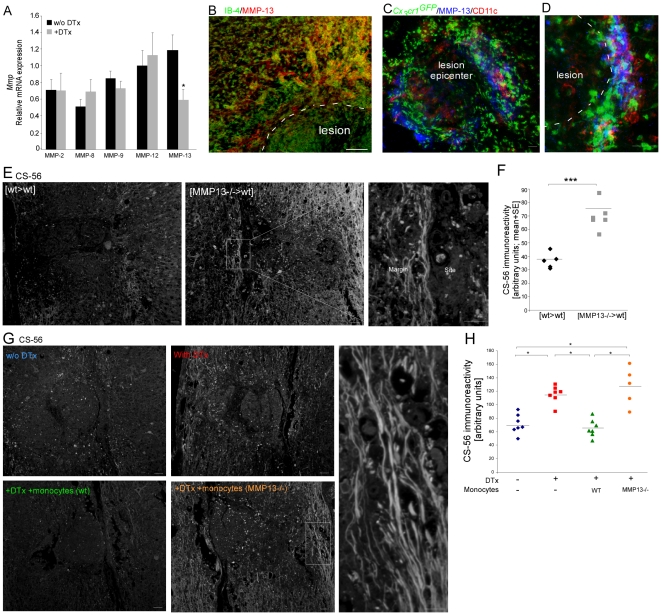
Infiltrating monocytes resolve glial scar matrix accumulation via the production of matrix metalloproteinase 13. (**A**) Analysis of expression of various *Mmp* genes in excised spinal cord tissues of [*CD11c*-DTR:*Cx_3_cr1* ^GFP/+^>wt] BM chimeras, with or without DTx treatment. (**B–D**) Immunohistochemical labeling of the injured spinal cord sections of [*Cx_3_cr1* ^GFP/+^>wt] BM chimeric mice for MMP-13, together with IB-4 (B), or GFP and CD11c (C; D). (**E,F**) [wt>wt] or [MMP-13^−/−^>wt] BM chimeras were subjected to spinal cord injury 8 weeks following transplantation, and analyzed 14 days post trauma for CSPG immunoreactivity. Representative pictures are presented in E. Quantification of CS-56 (CSPG) immunoreactivity in 2 mm^2^ sections, including the lesion site margin and surrounding parenchyma is shown in F. Deficiency in MMP-13 resulted in increased accumulation of CSPG *(Student's t-test*; ***p = 0.0002). (**G,H**) [*CD11c*-DTR>wt] BM chimeric mice were subjected to spinal cord injury 8 weeks following BM transplantation. Four groups were used: one group left untreated, one group was treated with DTx alone, and the other two groups received DTx in parallel to transfer with DTx-resistant monocytes isolated from either wt or MMP-13 KO mice; CSPG immunoreactivity was evaluated 14 days post injury. Representative pictures are shown in G. Quantification is shown in H (ANOVA; F_3,22_ = 15.4; p<0.0001). While reconstitution with wt monocytes restored the regulation of CSPG accumulation, MMP13^−/−^ monocytes failed to do so. Scale bar representation; 50 µm.

Matrix degradation enzymes have been suggested to mediate the tissue-remodeling properties of macrophages [32]. We next asked whether this crucial matrix-resolving function of the entering monocytes is mediated via the regulation of the matrix-degrading enzymes, matrix metalloproteinases (MMPs). While the majority of the matrix degrading enzymes tested showed increased levels following injury (**Figure S4**), only the matrix metalloproteinase (MMP)-13 was negatively affected as an outcome of the depletion of the monocyte-derived cells by DTx, as evaluated at day 5 post injury by RT-PCR of tissue spinal cord samples (**Fig. 4A**). Immunostaining of spinal cord sections confirmed that CD11c^+^ monocyte-derived MΦ that localized to the lesion margins are a major source of MMP-13 (**Fig. 4B–D**). MMP-13 showed highly restricted expression around the lesion site. This location at the margins of the injury site appears to be ideal for mediating glial scar-matrix degradation.

We next tested if MMP-13 expression by the infiltrating monocytes is essential for their scar remodeling capacity. To this end, we took advantage of MMP-13 knockout (KO) mice [35]. We first used [MMP-13^−/−^>wt] BM chimeras, in which the host BM is replaced with BM isolated from the knockout mice. In the resulting chimeras, the hematopoietic lineage is MMP-13 deficient, while the CNS tissue is of host (wild-type) origin. These mice were subjected to spinal cord injury 8 weeks post BM transplantation. Comparative analysis for CSPG immunoreactivity in spinal cord sections 14 days post trauma revealed higher CSPG accumulation in the MMP-13 KO chimeras (**Fig. 4E,F**). To further attribute this functionality to MMP13 expression by monocytes, we used the depletion-restoration strategy. DTx-depleted [*CD11c*-DTR>wt] BM chimeras, were replenished with CD115^+^ monocytes (via intravenous administration on d0 and d3 post injury), isolated from either wild-type mice, or from MMP-13 KO mice. Non-DTx treated chimeric mice served as a control. The mice were tested for CSPG immunoreactivity 14 days post injury. As reported above (**Fig. 3H,I**), monocyte depletion via DTx treatment resulted in increased CSPG accumulation, whereas reconstitution with wt monocytes led to a reduction in CSPG levels relative to the non-reconstituted mice (**Fig. 4G,H**). Importantly, while reconstitution with wt monocytes restored the regulation of CSPG accumulation, replenishment with MMP-13 KO monocytes failed to do so (**Fig. 4G,H**). Altogether, these results highlight the importance of monocytes as critical regulators of scar deposition, in particular its extracellular matrix CSPG, via the expression of the matrix degradation enzyme, MMP-13.

### Production of MMP-13 by monocytes is essential for the functional recovery from spinal cord injury

**Figure 5:**
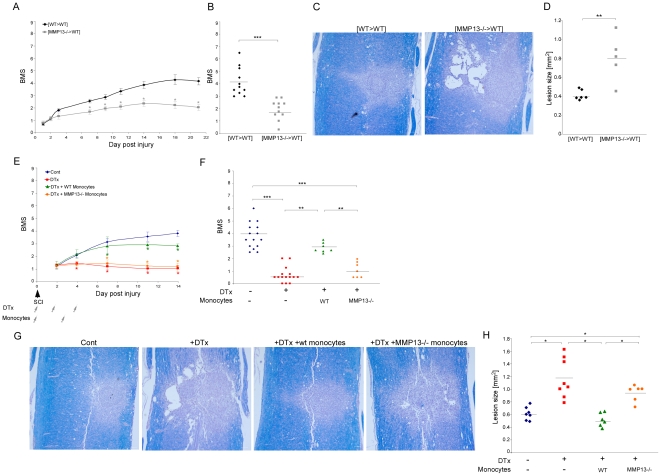
Expression of matrix metalloproteinase 13 by infiltrating monocytes is essential for functional recovery from spinal cord injury. (**A–D**) 45D-vaccinated, [wt>wt] or [MMP-13^−/−^>wt] BM chimeras were subjected to spinal cord injury 8 weeks post transplantation. (A,B) Motor function evaluation was performed according to the BMS. Follow up is shown in A and individual scorings at day 21 are shown in B. Deficiency in MMP-13 resulted in worse motor function (A-*Repeated ANOVA*; F_between-groups_(1,16) = 13.4; p<0.0001; B- *Student's t-test*; ***p<0.001). (C,D) Representative pictures of lesion sites stained for myelin integrity by Luxol-Nissl are presented in C. Lesion size evaluation according to Luxol-Nissl staining is shown in D. Increased lesion size is observed in MMP-13 deficient chimeras (*Student's t-test*; **p = 0.004). (**E–H**) [*CD11c*-DTR>wt] BM chimeric mice were subjected to spinal cord injury 8 weeks post BM transplantation. Four groups were used: one group was left untreated, one group was treated with DTx alone, and the other two groups received DTx in parallel to transfer with DTx-resistant monocytes isolated from either wt or MMP-13 KO mice. (E,F) Motor function evaluation was performed according to the BMS. Follow-up is shown in E and individual scorings at day 14 are shown in F. DTx depletion resulted in worse recovery. While reconstitution with wt monocytes restored lost motor function, replenishment with MMP-13 KO monocytes failed to do so (E-*Repeated ANOVA*; F_between-groups_(3,44) = 16.28; p<0.0001; F- *ANOVA*; F_3,39_ = 44.15; p<0.0001). (G,H) Representative pictures of lesion sites stained for myelin integrity by Luxol-Nissl, G. Lesion size evaluation according to Luxol-Nissl staining is shown in H (*ANOVA*; F_3,25_ = 15.6; p<0.0001). Scale bar; 100 µm.

In light of the tissue remodeling function attributed here to the infiltrating monocytes, together with the well-established phenomenon that CSPG degradation augments functional recovery following spinal cord injury [3], [5], [9], we next aimed to test if the remodeling property of these cells has functional implications to the repair process. We therefore repeated the experiments in the MMP-13 deficient mice, as described above, while evaluating the functional motor recovery of the hind limbs following spinal cord contusion according to the Basso Mouse Scale (BMS). In this non-linear scale, 0 represents complete paralysis of the hind limb, while a score of 9 represents normal mobility [36]. Mice in which the hematopoietic lineage lacked MMP-13, [MMP-13^−/^>wt], had worse motor function recovery of the hind limbs, relative to their control [wt>wt] chimeras (**Fig. 5A,B**). Evaluation of lesion size according to myelin staining, using Luxol-Nissl, further confirmed these results (**Fig. 5C,D**). To attribute this essential function to the monocyte subset, we again employed the depletion-restoration strategy; [*CD11c*-DTR>wt] BM chimeric mice were subjected to spinal cord injury 8 weeks post transplantation. Four groups were used: one group was left untreated, one group was treated with DTx, and the other two groups received DTx and passive transfer of DTx-resistant monocytes isolated either from wt or from MMP-13 KO mice, as described above. The mice were followed for motor function performance of the hind limbs, and scored according to the BMS. DTx depletion of monocyte-derived cells resulted in worse motor function performance following spinal cord injury, while reconstitution of the depleted mice with monocytes resistant to the toxin, restored the lost motor function (**Fig. 5E,F**). In contrast, replenishment of the monocyte pool with MMP-13-deficient monocytes failed to restore recovery, and resulted in similar motor function as that observed in the DTx-treated mice that did not receive monocytes (**Fig. 5E,F**). Evaluation of lesion size confirmed these results (**Fig. 5G,H**). These results attribute a critical functional relevance to the infiltrating monocytes via the expression of the matrix remodeling enzyme MMP-13.

Importantly, these two characteristics of the infiltrating monocytes following spinal cord injury, the anti-inflammatory nature described by us before [14] and the scar degradation property identified here, are not contradictory. These two properties should be viewed as two complementary aspects of their resolving phenotype. These cells are endowed with a panel of properties to resolve the first phase of the dynamic response post injury, which is characterized by both intense inflammation and scar formation. In fact, the connection between the capacity of monocytes to remodel cellular matrix and promote regeneration and between their anti-inflammatory essential properties was suggested previously in peripheral tissue healing [18], [19]. Interestingly, *in vitro* cultures of naïve monocytes revealed enhanced expression of the *Mmp13* transcript when the cells were grown on a CSPG substrate (relative mRNA expression; 0.5 on PDL vs. 2.5 on CSPG), raising the possibly of an endogenous feedback loop, in which the glial scar matrix induces its own degradation.

## Discussion

**Figure 6:**
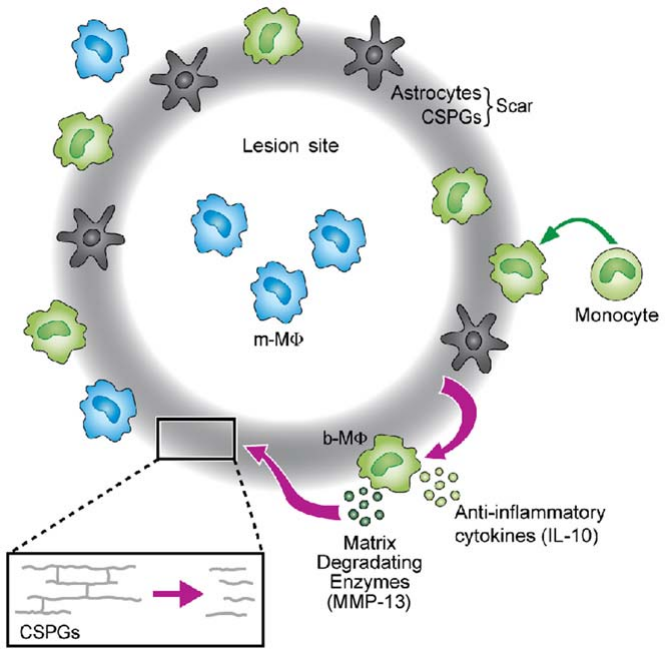
Schematic illustration of the mutual relationship between the resolving monocytes and the glial scar matrix. The glial scar matrix serves as a regulatory template, dictating the anti-inflammatory nature of the infiltrating monocytes that encounter it. In turn, the monocytes that used the scar for their own education regulate the scar degradation and resolution via the secretion of matrix degradation enzyme, MMP-13.

In this study, we demonstrated that two main phenomena that occur in the injured CNS, the inflammatory response and accumulation of glial scar, which were generally assumed to be independent and separately detrimental, are in fact tightly connected in an intimate relationship that promotes their mutual potential to benefit healing (**Fig. 6**; scheme). The glial scar matrix was found here to serve as a necessary scaffold, skewing monocytes towards the resolving phenotype, characterized by the production of the anti-inflammatory cytokine IL-10, thereby promoting resolution (termination) of the local inflammation. In a reciprocal relationship, the monocyte-derived cells were found to produce the matrix degrading enzyme MMP13 and thereby regulate CSPG accumulation and promote repair.

The immunosuppressive nature of CSPG in the response to trauma, as observed here, is consistent with data demonstrating that scar-associated astrocytes are required to maintain a balanced inflammatory response [37], [38]. The immunoregulatory nature of this matrix molecule is substantiated by our previous observation that it promotes neurotrophic factor production by the resident microglia [9]. In addition, the immunoregulatory features of the scar appear to be a general feature of tissue healing, as proteoglycans are key immune-modulators following trauma to internal organs [8]. Such a function performed by the matrix is essential under the unfavorable milieu that exists at the site of trauma, which is laden with factors known to mediate M1 skewing. As the extracellular matrix around the site is a complex structure, built like a branched tree, it seems that every component within it has a dramatic effect on the local organization of the perineuronal network created around the lesion site following injury. We can suggest that this complex structure can, on the one hand, have a direct effect on the encountering cells, and on the other hand serves as a physical barrier isolating these cells from the material concentrated at the epicenter, which possesses M1-skewing properties.

Scar deposition is an essential response to the trauma, that should be tightly controlled [9]; although it is essential for the repair at the acute phase [9], the scar becomes an obstacle in the subsequent steps [3], [5], [9]. Such timely regulation of scar deposition is shown here to be achieved by the bi-directional interaction between the glial scar and the monocytes; MΦ, which use the scar for their own education, were identified here as the cellular component that promotes scar degradation via the production of matrix degradation enzymes. In support of our *in vivo* observation, *in vitro* skewing of macrophages towards an M2 phenotype was recently shown to promote axonal regeneration [11]. In addition, scar resolving properties were recently ascribed to MΦ in the resolution of a different kind of collagen-based scar, during hepatic fibrosis, in which the same MMP described here, MMP-13, plays a fundamental role, as well [32]. MMP-13 was not previously proposed to have a role in spinal cord repair. Only recently was it suggested, based on *in vitro* studies, to degrade CSPG. In addition to CSPG, MMP-13 may regulate other components of the perineuronal net, such as Tenascin and Aggrecan, which are known to be substrates for this enzyme and critical components of the glial scar, further highlighting its functional relevance to the dynamic repair response post trauma. Interestingly, tissue matrix modulation and regeneration properties were recently attributed to monocyte-derived macrophages, and more specifically to the anti-inflammatory subset [18], [19]. Thus resolution/termination of inflammation and tissue remodeling are tightly interconnected, and seem to be a general property of wound healing macrophages. In fact, one can view the two essential characteristics of the infiltrating monocytes for the recovery from spinal cord injury, the anti-inflammatory properties described by us before [14] and scar degradation properties of these cells revealed here, as two aspects of their resolving phenotype. These cells ‘resolve’ the first phase of the dynamic response to the injury, which is characterized by both intense inflammation and scar formation.

The identified monocyte-glial scar interplay thus primes the resolution phase of CNS tissue healing, thereby providing a platform for the repair response. Revealing the underlying mechanism behind this essential dialogue might enable the development of novel therapeutic approaches to fine-tune it. The recognition of a novel enzyme that modulates CSPG deposition and has a fundamental contribution to the repair process indicates a potential target for future therapies. Our findings harbor significant clinical implications not only for the repair of CNS injuries, but also for the resolution of autoimmune diseases of the CNS, in which inflammation goes awry. In addition, as regulatory MΦ/MDSCs provide a predominant tolerance mechanism by which tumors escape the immune system, a deeper understanding of how monocytes are skewed by matrix components might suggest additional therapeutic avenues.

## Materials and Methods

### Animals

Six types of mice were used: (1) C57BL/6J mice; (2) CD45.1 mice (carrying an allotypic marker, CD45.1); (3) heterozygous mutant *Cx_3_cr1* ^GFP/+^ mice (B6.129P- *Cx_3_cr1*tm1Litt/J), in which one of the *Cx3cr1* chemokine receptor alleles is replaced with a gene encoding GFP [green fluorescent protein] [26]; (4) *CD11c*-DTR transgenic mice (B6.FVB-Tg Itgax-DTR/GFP 57Lan/J), carrying a transgene encoding the human diphtheria toxin receptor [DTR] under control of the murine *CD11c* promoter [34]; *Cx_3_cr1* ^GFP/+^ and *CD11c*-DTR transgenic mice were a generous gift from Prof. Steffen Jung. (5) *CD11c*-DTR: *Cx_3_cr1* ^GFP/+^ transgenic mice (heterozygous for both the *Cx_3_cr1* ^GFP^ locus and the *CD11c*-DTR transgene); and (6) MMP-13 knockout mice [35], a generous gift from Prof. Carlos López-Otín. For all experiments, adult males aged 8–10 weeks were used. Animals were supplied by the Animal Breeding Center of The Weizmann Institute of Science. All animals were handled according to the regulations formulated by the Institutional Animal Care and Use Committee (IACUC).

### Bone Marrow Radiation Chimeras

[*Cx_3_cr1* ^GFP/+^>wt], [*CD11c*-DTR>wt], [MMP-13^−/−^>wt], [wt>wt] and [*CD11c*-DTR:*Cx_3_cr1* ^GFP/+^>wt] BM chimeras were prepared by subjecting gender-matched recipient mice (8–10 week old) to lethal whole-body irradiation (950 rad) while shielding the brain, as previously described [9], [14]. The mice were then reconstituted with 3–5×10^6^ BM cells harvested from the hind limbs (tibia and femur) and forelimbs (humerus) of the appropriate donor mice. BM cells were obtained by flushing the bones with Dulbecco's PBS under aseptic conditions, and were then collected and washed by centrifugation (10 min, 1,000 rpm, 4°C). The chimeric mice were subjected to spinal cord contusion 8–10 weeks after BM transplantation.

### Spinal Cord Injury

The spinal cords of deeply anesthetized mice were exposed by laminectomy at T12, and contusive (200 kdynes) centralized injury was performed using the Infinite Horizon spinal cord impactor (Precision Systems), as previously described [9], [14]. The animals were maintained on twice-daily bladder expression. Animals that were contused in a nonsymmetrical manner were excluded from the experimental analysis.

### Xyloside Treatment

Xyloside (4-methylumbelliferyl-b-D-xylopyranoside; Sigma-Aldrich) was injected as previously described (0.8 mg/mouse) [9]. Briefly, the mice were intraperitoneally (IP) injected twice daily for 5 consecutive days, starting either immediately after the injury or 2 days later. For histological analysis, mice were killed 7 days or 14 days after the injury.

### Diphtheria Toxin Administration

Diphtheria toxin (DTx; 8 ng/g body weight; Sigma) was injected intraperitoneally (IP), repeatedly at 1 day intervals, starting immediately after the injury.

### Adoptive Transfer of Monocytes

CD115^+^ monocytes were isolated as previously reported [39]. Briefly, BM cells were harvested from the femora and tibiae of naïve mice, and enriched for mononuclear cells on a Ficoll density gradient. The CD115^+^ BM monocyte population was isolated through MACS enrichment using biotinylated anti-CD115 antibodies and streptavidin-coupled magnetic beads (Miltenyi Biotec) according to the manufacturer's protocols. Following this procedure, monocytes (purity 90%) were intravenously (IV) injected (3.5×10^6^ cells per mouse) twice during the first week of recovery, on d0 and d3 post injury.

### Immunohistochemistry

Due to technical limitations of some of the antibodies that were used, two different tissue preparations (paraffin embedded and microtomed frozen sections) were used, as previously described [9]. Whenever possible, the results were confirmed using both techniques. The following antibodies were used: rabbit anti-GFP (1∶100; MBL), rabbit anti-glial fibrillary acidic protein (GFAP; 1∶100; Dako Cytomation), goat anti-IL-10 (1∶20; R&D Systems), mouse anti-CS-56 (1∶100; Sigma), mouse anti-Hu (1∶50; Rhenium), mouse anti-MMP13 (1∶50; Abcam), and hamster anti-CD11c (1∶50; Chemicon). For microglial/MΦ labeling, TRITC- or FITC-conjugated *Bandeiraea simplicifolia* isolectin B4 (IB-4; 1∶50; Sigma-Aldrich) was added for 1 h to the secondary antibody solution. Secondary antibodies used included: Cy2-conjugated donkey anti-rabbit antibody, Cy2/Cy5 conjugated donkey anti-mouse antibody, Cy3-conjugated donkey anti-mouse, Cy3-conjugated donkey anti-goat, and biotin goat anti-hamster (1∶200; all from Jackson Immuno Research). Cy3-streptavidin was used for CD11c staining. The slides were exposed to Hoechst stain (1∶4,000; Invitrogen Probes) for 1 min. GFAP staining was used for demarcation of the lesion site.

Myelin integrity was qualitatively and quantitatively examined on paraffin-embedded sections that were stained with Luxol fast blue for myelin, and with Nissl to identify the nuclei and the thin cytoplasmic layer around them. Myelin phagocytosis was analyzed in sections stained with Oil Red O (Fisher Scientific) and counterstained with Mayer's hematoxylin to identify cell nuclei, as previously described [10], [31]. For microscopic analysis, a Nikon fluorescent microscope (Nikon E800) or Zeiss LSM 510 confocal laser scanning microscope were used. Longitudinal sections of the spinal cord were analyzed. Numbers of cells, immunoreactivity (density) and lesion size were all determined automatically with *Image-Pro* Plus 4.5 software (Media Cybernetics). To measure lesion size, demarcation of the damaged site was determined according to Luxol-Nissl staining as well as H&E staining. In order to avoid overestimation due to counting of partial cells that appeared within the section, we took special care to count only cells with intact morphology and a nucleus that was larger than 4 µm in diameter, both in the manual and software-automated counting. The *ImagePro* quantification was performed using 2 mm^2^ spinal cord tissues pictures, centralized on the lesion site, which included lesion site, the margins and surrounding undamaged parenchyma. Three sections from different depths were assessed for each animal, and 4–8 mice were tested in each group. For immunoreactivity measurements, the values are presented in arbitrary units and indicate total reactivity in the tissue. The number of cells per mm^3^ was calculated by considering the thickness of the sections.

### Isolation of Spinal Cord Cells and Flow Cytometric Analysis

Mice subjected to spinal cord injury were killed by an overdose of anaesthetic and their spinal cords were prepared for flow cytometric analysis by perfusion with PBS via the left ventricle. Spinal cord sections were cut from individual mice, including the injured site and adjacent margins (4 mm long in each of the sections), and tissues were homogenized using a software controlled sealed homogenization system (Dispomix; http://www.biocellisolation.com). For IL-10 staining, 2×10^6^/ml cells were cultured on 96-well plates. The following fluorochrome-labeled monoclonal antibodies were purchased from BD Pharmingen, BioLegend, or eBioscience and used according to the manufacturers' protocols: PE conjugated anti-CD11b, IL-10 and CD115 antibodies, and allophycocyanin-conjugated anti-CD45.1, and CD11b antibodies. Cells were analyzed on a FACSCalibur cytometer (BD Biosciences) using CellQuest software (BD Biosciences) or on a LSRII cytometer (BD Biosciences) using Flow Jo software (Tree Star). Isotype controls were routinely used in all the experiments. In addition, in each experiment, relevant negative control groups were used to identify the populations of interest and to exclude others.

### Multiplex cytokine analysis system

Wild-type C57BL/6J injured and non-injured mice were killed at different time points after spinal cord injury. Samples from lesion sites (4 mm length of spinal cord tissue, including lesion site, margins and surrounding non-injured parenchyma) were pooled in groups of three. The excised tissues were homogenized in PBS containing protease inhibitors (1∶100; P8340, Sigma). Four freeze-thaw cycles were performed to break the cell membranes (3 minutes each). Homogenates were then centrifuged for 10 min at 500 *g*, and the total protein quantities in supernatants were determined by Bradford reagent. Frozen supernatants were assayed in duplicate using Multiplex Bead-based Luminex Assays (MILLIPLEX mouse cytokine/chemokine panel or TGFβ1,2,3 MILLIPLEX kit; Millipore), performed by outsourcing (American Medical Laboratories), according to the manufacturer's instructions. Results are expressed as picograms of protein per milligram of total tissue protein.

### Assessment of functional recovery from spinal cord injury

Recovery was evaluated by hind-limb locomotor performance, assessed according to the open-field Basso Mouse Scale (BMS) [36], with nonlinear scores ranging from 0 (complete paralysis) to 9 (normal mobility); each score represents a distinct motor functional state. Mice were randomly separated into groups, while verifying that the average starting score was similar in all groups. Blind scoring ensured that observers were not aware of the treatment received by each mouse. Locomotor activity in an open field was monitored twice a week by placing the mouse for 4 min at the center of a circular enclosure (diameter 90 cm, wall height 7 cm) made of molded plastic with a smooth, non-slippery floor. Before each evaluation, the mice were carefully examined for peritoneal infection, wounds in the hind limbs, and tail and foot autophagia. Animals that showed a difference of more than 2 score points between their two hind limbs were excluded from the experimental analysis. The results showing functional outcomes presented in this study were, in each case, from a single experiment representative of several independent replicates, as indicated in the figure legends. As spontaneous recovery from spinal cord injury is limited, we used the previously described [9], [14] protocol of 45D vaccination (100 µg; emulsified in an equal volume of complete Freund's adjuvant containing *Mycobacterium tuberculosis* (2.5 mg/ml; Difco); 1 week prior the injury), which creates a more sensitive system to evaluate functional recovery.

### Culture of monocytes

25 cm^2^ Falcon tissue culture flasks (BD Biosciences) were coated either with poly-D-lysine (PDL) (20 µg/ml; Sigma-Aldrich) in borate buffer, pH 8 for 4 h; or CSPG (10 µg/ml, Sigma-Aldrich) in PBS. CD115^+^ cells were isolated as described above. A total of 6×10^6^ CD115^+^ cells were seeded per flask on CSPG or on the control substrate, PDL, in the following media: RPMI-1640 (Biological Industries, Beit Ha-Emek, Israel), 10% FCS, 2 mM L-Gln, 100 U/ml penicillin, and 100 µg/ml streptomycin, NAA, and 1 mM sodium pyruvate. The purified cells were cultured in 5% CO_2_ at 37°C. The cultures were harvested 2 or 5 days later, and the supernatants collected. IFN-γ was added to some of the cultures (100 ng/µl), as indicated.

### Quantitative Real time PCR

Target cells or tissue were homogenized in Tri reagent (Sigma), and total RNA was extracted using Qiagen RNeasy Mini-kit. Random hexamers (AB) were used for first-strand cDNA synthesis. Both procedures were performed according to the manufacturer's instructions. The relative amounts of mRNA were calculated by using the standard curve method, and were normalized to the housekeeping gene, peptidylprolyl isomerase A (PpiA). Each RNA sample was run in triplicate, and each group was comprised of three to five animals. The primers for all genes tested (see Table 1) were designed using PrimerQuest software, from Integrated DNA Technologies (http://eu.idtdna.com).

**Table 1:** 

	Forward Primer	Reverse Primer
*PpiA*	AGCATACAGGTCCTGGCATCTTGT	CAAAGACCACATGCTTGCCATCCA
*Il10*	TGAATTCCCTGGGTGAGAAGCTGA	TGGCCTTGTAGACACCTTGGTCTT
*Mmp2*	TCTGGTGCTCCACCACATACAACT	ATTGCCACCCATGGTAAACAAGGC
*Mmp8*	GGAATCCTTGCCCATGCCTTTCAA	TGTCCAAATTCATGAGCAGCCACG
*Mmp9*	AGACGTGGGTCGATTCCAAACCTT	TCGCGGCAAGTCTTCAGAGTAGTT
*Mmp12*	AAAGGTGGTACACTAGCCCATGCT	GCAACAAGGAAGAGGTTTGTGCCT
*Mmp13*	TTCTTGTTGAGCTGGACTCCCTGT	TGCTCTGCAAACACAAGGTCTTCC

### ELISA assay for cytokine levels

Cytokine ELISA for IL-10 was performed on culture supernatants of the *in vitro* experiment, according to the manufacturer's instructions (eBioscience, Mouse Interleukin-10 Ready-SET-Go!). Each supernatant sample was run in triplicate, and a total of five supernatants were used per group. Results were expressed as picograms of protein per milliliter of supernatant.

### Statistical Analysis

Data were analyzed using the Student's *t*-test to compare between two groups. One-way ANOVA was used to compare several groups; the Tukey's HSD procedure (*p* = 0.05) was used for follow-up pairwise comparison of groups. Repeated measures ANOVA was used in the functional BMS scoring with follow-up comparison of treatments for each day by contrast *t*-test and correction for multiple comparison by the Holm method (*p* = 0.05). The specific tests used to analyze each set of experiments are indicated in the figure legends. The results are presented as mean ± SE. In the graphs, *y*-axis error bars represent SE.

## Supporting Information

Figure S1 **Kinetic evaluation of IL-10 expression at the lesioned spinal cord.** Spinal cord sections were isolated at different time points following injury and immunostained for IL-10 (red) and GFAP (green). Non injured sections were also co-stained for the neuronal marker, Hu (green), and the cytokine, IL-10 (red). Scale bar; 50 µm.
(TIF)

Figure S2 **Delayed administration of xyloside does not disrupt the spatial organization nor the resolving phenotype of the infiltrating monocytes.** (**A, B**) Immunohistochemical staining of [*Cx_3_cr1* ^GFP/+^>wt] BM chimeric mice treated with xyloside at later stages, with (A) CSPG (red) and GFP (green), or (B) IL-10 (red) and GFAP (green). (**C**) Quantitative analysis of IL-10 immunoreactivity (left panel, *Student's t-test*; p = 0.43) and number of IL-10 expressing cells (right panel, *Student's t-test*; p = 0.968). (**D**) Quantification of activated microglia/MΦ according to IB-4 immunoreactivity (*Student's t-test*; *p = 0.037). Scale bar; 50 µm. *y*-axis error bar represents SEM.
(TIF)

Figure S3 **Myelin engulfment does not correlate with the resolving phenotype of macrophages at the lesioned spinal cord.** Oil Red O staining of spinal cord tissues isolated 7 days post injury, from mice treated with PBS or xyloside for 5 consecutive days immediately post injury. Equal distribution of Oil Red O staining was seen at the lesion center and its margins. No significant differences could be observed between the groups. Scale bar; 100 µm.
(TIF)

Figure S4 **Matrix metalloproteinase gene expression levels following insult.** Analysis of expression of various MMP genes in excised spinal cord tissues by RT-PCR at different time points following the insult. The relative expression levels are presented. (*Mmp12*; ANOVA; F_4,13_ = 44.7; *p* = 0.0005. *Mmp13*; ANOVA; F_4,11_ = 15.4; *p* = 0.0075; *Mmp8*; ANOVA; F_4,11_ = 16.98; *p* = 0.0053. *Mmp9*; ANOVA; F_4,11_ = 1.6; *p* = 0.239. *Mmp2*; ANOVA; F_4,15_ = 62.76; *p*<0.0001). Asterisks indicate significant differences compared to non-injured animals. *Y* axis error bar represents SEM.
(TIF)
