# Supplementary figures and images for: The Glial Scar-Monocyte Interplay: A Pivotal Resolution Phase in Spinal Cord Repair
Source: PLoS One. 2011 Dec 21;6(12):e27969. doi: 10.1371/journal.pone.0027969 (PMC3244386; doi:10.1371/journal.pone.0027969)

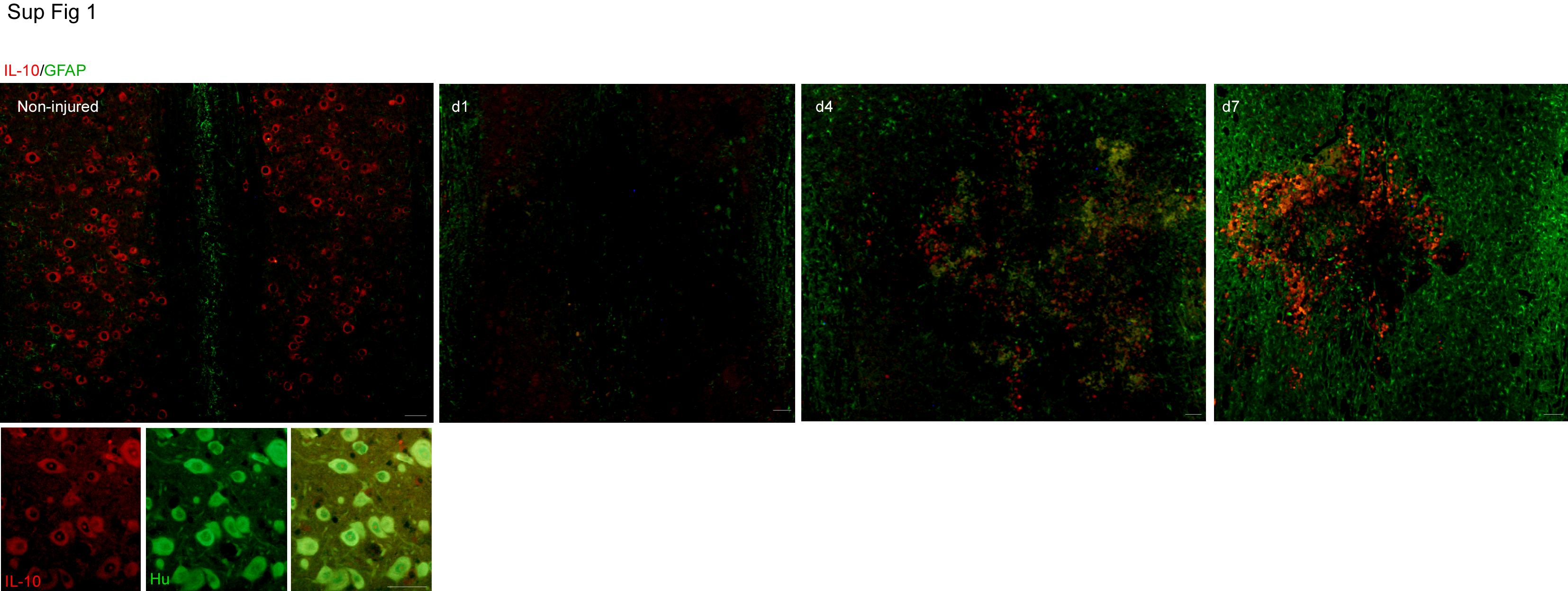

Supplement: Figure S1 — Kinetic evaluation of IL-10 expression at the lesioned spinal cord. Spinal cord sections were isolated at different time points following injury and immunostained for IL-10 (red) and GFAP (green). Non injured sections were also co-stained for the neuronal marker, Hu (green), and the cytokine, IL-10 (red). Scale bar; 50 µm. (TIF) [file pone.0027969.s001.tif]

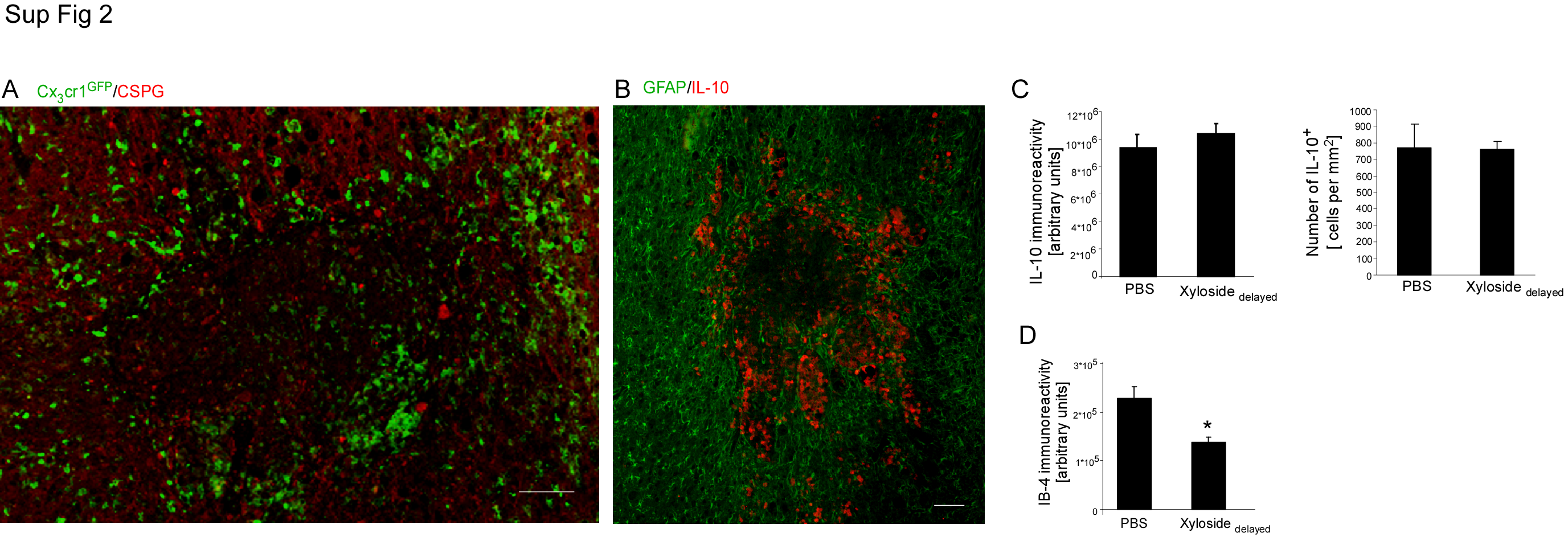

Supplement: Figure S2 — Delayed administration of xyloside does not disrupt the spatial organization nor the resolving phenotype of the infiltrating monocytes. (A, B) Immunohistochemical staining of [Cx3cr1 GFP/+>wt] BM chimeric mice treated with xyloside at later stages, with (A) CSPG (red) and GFP (green), or (B) IL-10 (red) and GFAP (green). (C) Quantitative analysis of IL-10 immunoreactivity (left panel, Student's t-test; p = 0.43) and number of IL-10 expressing cells (right panel, Student's t-test; p = 0.968). (D) Quantification of activated microglia/MΦ according to IB-4 immunoreactivity (Student's t-test; *p = 0.037). Scale bar; 50 µm. y-axis error bar represents SEM. (TIF) [file pone.0027969.s002.tif]

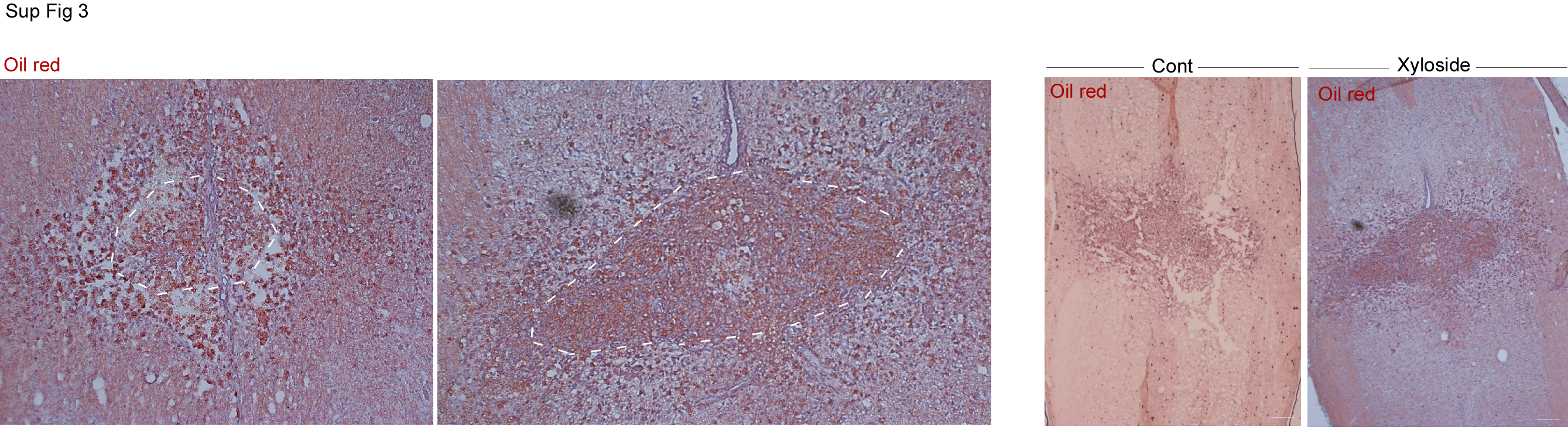

Supplement: Figure S3 — Myelin engulfment does not correlate with the resolving phenotype of macrophages at the lesioned spinal cord. Oil Red O staining of spinal cord tissues isolated 7 days post injury, from mice treated with PBS or xyloside for 5 consecutive days immediately post injury. Equal distribution of Oil Red O staining was seen at the lesion center and its margins. No significant differences could be observed between the groups. Scale bar; 100 µm. (TIF) [file pone.0027969.s003.tif]

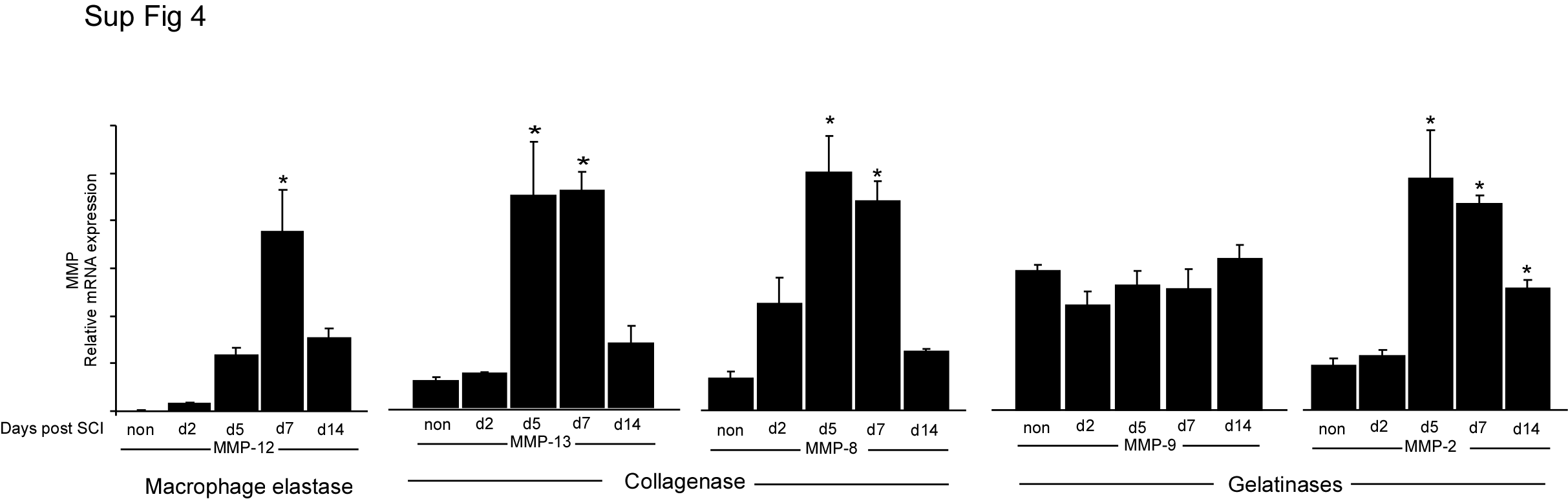

Supplement: Figure S4 — Matrix metalloproteinase gene expression levels following insult. Analysis of expression of various MMP genes in excised spinal cord tissues by RT-PCR at different time points following the insult. The relative expression levels are presented. (Mmp12; ANOVA; F4,13 = 44.7; p = 0.0005. Mmp13; ANOVA; F4,11 = 15.4; p = 0.0075; Mmp8; ANOVA; F4,11 = 16.98; p = 0.0053. Mmp9; ANOVA; F4,11 = 1.6; p = 0.239. Mmp2; ANOVA; F4,15 = 62.76; p<0.0001). Asterisks indicate significant differences compared to non-injured animals. Y axis error bar represents SEM. (TIF) [file pone.0027969.s004.tif]
